# Editorial: Mechanisms of cell death in acute liver diseases and the pathobiology of sterile inflammation: The double-edged sword problem

**DOI:** 10.3389/fimmu.2026.1792869

**Published:** 2026-02-03

**Authors:** Juan C. Cutrin, Carmen Peralta Uroz

**Affiliations:** 1 Molecular Biotechnology Center II “Guido Tarone”, Department of Molecular Biotechnologies and Sciences for the Health, University of Torino, Torino, Italy; 2 Protective strategies against hepatic ischemia reperfusion injury Research Group, Institute of Biomedical Investigations August Pi i Sunyer (IDIBAPS), Barcelona, Spain

**Keywords:** acute liver pathologies, biomarkers, cell death, sterile inflammation, therapeutics

The liver is a dynamic metabolic and immune organ that is highly vulnerable due to continuous internal and external challenges. These challenges include viral infections, alcohol consumption, drugs, xenobiotics, metabolic disturbances, traumatic injuries, and post-ischemic damage as occurs during transplantation (1, 2).

Such stressors can trigger different types of cell death (CD): a) uncontrolled, like onco-necrosis and/or b) regulated, like apoptosis, pyro-ptosis, necroptosis and ferroptosis (3). Importantly, these forms of CD are not only limited to hepatocytes, but they also involve non -parenchymal cells creating a crosstalk among both cells populations that significantly impact the liver’s complex functions (1–3).

Moreover, it was observed that these different CD modes can coexist simultaneously in pathological contexts (*i.e.*, onco-necrosis + apoptosis + ferroptosis in ischemia-reperfusion injury) and several of them share overlapping mechanisms that can act as a “backup” death strategy. Thus, the various CD forms are not completely independent or compartmentalized but instead different CD ways show relevant crosstalk (4).

The surge in dying cells results in an overwhelming presence of cell debris and the release of damage-associated molecular patterns, also known as “alarmins”. The release of alarmins by the liver is associated with loss of tissue homeostasis. The turn loose and recognition of these alarmins by specific pattern-recognition receptors triggers an inflammatory response, when properly regulated serves as a damage-control mechanism aimed at restoring homeostasis through tissue repair and liver regeneration. In contrast, a dysregulated or overt sterile inflammation response can inadvertently cause further organ damage, potentially resulting to liver failure and extrahepatic tissue damage (5, 6).

Our comprehension of CD pathways as initiators and modulators of disease is continually growing. Many CD pathways can coexist simultaneously, and the release of various alarmins can elicit diverse responses depending on the cell types. Moreover, cells undergo multiple regulated cell death (RCD) procedures through a wide range of crosstalk that can be activated simultaneously under specific conditions, a fact that is consistent with the recently proposed concept of PANoptosis (“P,” pyro- ptosis, “A,” apoptosis; “N,” necroptosis). PANoptosis is a modality of inflammatory RCD mediated by PANoptosome complexes with key features of pyro-ptosis, apoptosis, and/or necroptosis, which cannot be explained by any of the three RCD mechanisms alone (7–12). Understanding how CD pathways operate under different pathological situations remains to be elucidated.

In this Research Topic, the authors have provided contributions about crucial aspects of CD mechanisms and inflammation in acute liver pathologies: 1) The CD mechanisms in liver transplantation, 2) The Phase angle (PhA) of the bioelectrical impedance as biomarker for evaluating acute on chronic liver failure (ACLF), 3) Copper metabolism and cuproptosis, and 4) The inter-relationship between metabolic dysfunction-associated liver disease (MASLD) and systemic arterial hypertension (SAH) and the key mechanism of inflammation.

Hirao et al. examined the various CD mechanisms that influence liver graft outcomes, including apoptosis, necrosis. Autophagy, and non-apoptotic inflammatory CD. The authors discuss how these ways of CD are driven by ischemia-reperfusion damage, which contributes to graft dysfunction. Moreover, authors have uncovered novel non-apoptotic death pathways, such as necroptosis, pyropotosis, ferroptosis, panaptosis and netosis, that may also influence transplant outcome. Finally, authors analysed the growing use of machine perfusion technologies as a promising treatment for mitigating ischemia-reperfusion injury in liver transplantation.

Cai et al. analysed the problem of the acute hepatic decompensation on chronic liver failure, a critical syndrome defined by rapid hepatic deterioration with high short-term mortality and progressive extrahepatic failure, thereby necessitating prompt prognostic assessment. Author studied in 78 ACLF patients the prognostic significance of the PhA. Authors concluded that PhA is a readily measurable biomarker reflecting inflammatory status, that serves as a valuable indicator for assessing disease severety and predicting 90 days survival in ACLF.

In the paper of Chen et al, authors described the copper metabolism and how its homeostasis disruption can lead to a wide range of liver diseases. Specially, authors have focused on a form of programmed CD associated with high dysregulated levels of the ion, namely cuproptosis. Authors delved the role of cuproptosis in several liver pathologies: Wilson disease, alcoholic disease, non-alcoholic fatty liver disease, acute liver injury and hepatocellular carcinoma, as well as their therapeutic potential.

It is well known that MASLD sick patients have a high risk of developing SAH. Likewise, SAH sick patients show an increased susceptibility to MASLD suggesting a mutual pathogenic mechanism Solleiro-Villavicencio et al. in this review described the most significance features of the intricate and bidirectional interplay between both diseases with focus on inflammation as the central shared pathogenic mechanism. Authors highlighted the importance of investigating anti-inflammatory and immunomodulatory strategies as potential therapeutic approaches to slow the progression of both MASLD and SAH.

In conclusion: we now recognize several different forms of cell death, each executed by different pathways and mediators of sterile inflammation. The mechanisms are being unraveled and their contribution to human liver diseases is being carefully examined. These insights reveal further complexity in hepatic damage, but also provide new and promising therapeutic avenues for the treatment of human liver diseases.
